# Development and preliminary validation of a Korean version of the Personal Relative Deprivation Scale

**DOI:** 10.1371/journal.pone.0197279

**Published:** 2018-05-10

**Authors:** Hyunji Kim, Eunbee Kim, Eunkook M. Suh, Mitchell J. Callan

**Affiliations:** 1 Faculty of Psychology, Universität Wien, Vienna, Austria; 2 Department of Psychology, Yonsei University, Seoul, South Korea; 3 Department of Psychology, University of Bath, Bath, United Kingdom; Leibniz Institute for Educational Trajectory, GERMANY

## Abstract

The current research developed and validated a Korean-translated version of the Personal Relative Deprivation Scale (PRDS). The PRDS measures individual differences in people’s tendencies to feel resentful about what they have compared to what other people like them have. Across 2 studies, Exploratory Factor Analyses revealed that the two reverse-worded items from the original PRDS did not load onto the primary factor for the Korean-translated PRDS. A reduced 3-item Korean PRDS, however, showed good convergent validity. Replicating previous findings using Western samples, greater tendencies to make social comparisons of abilities (but not opinions) were associated with higher PRDS (Studies 1 and 2), and participants scoring higher on the 3-item Korean PRDS were more materialistic (Studies 1 and 2), reported worse physical health (Study 1), had lower self-esteem (Study 2) and experienced higher stress (Study 2).

## Introduction

Personal relative deprivation (PRD) refers to resentment caused by the awareness that one is deprived of desired and deserved outcomes compared to what others have [1–2]. People vary in terms of how much they experience PRD, and Callan, Shead, and Olson [3] developed the Personal Relative Deprivation Scale (PRDS) to gauge this individual difference. The PRDS is an important and unique predictor of a variety of beliefs and behaviours, including stronger gambling urges and increased problem gambling severity [4–5], higher materialism [6–7], increased delay discounting [8], poorer mental and physical health [9], and lower prosociality [10].

Although research has shown that the PRDS predicts a variety of important outcomes, with a few exceptions [7], most of this research has been conducted using Western samples. Does the PRDS predict theoretically relevant outcomes among people accustomed to Eastern cultural contexts? Some evidence suggests that people from Eastern (vs. Western) cultures might respond differently to adverse social comparisons due to lower individualism resulting in reduced emotional reactions to self-focused contexts [11]. Meanwhile, other research suggests that East Asians might nonetheless be concerned about what they have relative to others because of the constant self-other comparisons required to achieve group harmony and conform to group norms [12–13]. It is therefore not clear how PRD is associated with its known antecedents (e.g., social comparison tendencies) and consequences (e.g., increased stress) in Eastern cultures.

Among East Asian countries that show collectivistic characteristics is South Korea [14–15]. Several dimensions define different types of individualistic and collectivistic culture [16]. In particular, Korean collectivism is characterized by having verticality (vs. horizontality; i.e., People submit to the authorities of their in-group and show willingness to sacrifice their own benefit for the benefit of the in-group; [17]) with an emphasis on “relatedness” [18]. A greater tendency to think about oneself in relation to others could mean that Koreans might show a greater tendency to make social comparisons. Some indirect evidence supports this notion by showing that people with Asian background (e.g. Chinese, Korean, and Japanese descent) seek more upward social comparisons than people with European background (e.g., British, French, German descent; [13]). Given that PRD requires upward social comparison, PRD might play a crucial role in Korean population for explaining the relation between tendency to make social comparisons and theoretically relevant behaviors (i.e., health and materialism). Across two studies, we developed and tested a Korean-translated version of the PRDS to examine this idea.

## Study 1

The purpose of Study 1 was to (a) develop a Korean-translated version of Callan et al.’s [3] PRDS and (b) examine its associations with people’s tendencies to engage in social comparisons, self-rated physical health, personality, and materialism among a sample of Korean participants. The experience of PRD requires social comparison [2], but not all types of social comparisons are theoretically relevant to PRD. Using a Western sample, Callan, Kim, and Matthews [19] found that individual differences in people’s tendencies to engage in social comparisons of abilities (e.g., “I am not doing as well as other people”) positively predicted PRD, but social comparisons of opinions (e.g., “My beliefs are different from others’ beliefs”) did not. We examined whether this pattern would replicate in a sample of participants from an Eastern cultural background by measuring individual differences in people’s tendencies to engage in social comparisons along with PRD. Kim, Callan, Gheorghiu, and Matthews [6] also found that people higher in PRD tended to be more materialistic, and that the positive relationship between social comparisons of abilities and materialism was mediated by PRD. We therefore gauged participants’ materialism to examine whether these patterns would replicate in an Eastern cultural context. Finally, previous research with Western participants has shown that people higher in PRD tend to have worse health [9] and are lower in emotional stability and conscientiousness [3], so we included measures of self-rated physical health and personality to explore these associations in our Korean sample.

## Method

The studies included in the current paper were approved by the ethics committee of the department of Psychology at Yonsei University.

### Participants

Korean participants (*N* = 224; 56.3% male; *M*_*age*_ = 24.10, *SD*_*age*_ = 5.37) were recruited either for an exchange of course credits (*n* = 155) or via social media (*n* = 69). Participants filled out an online survey using a personal computer in a university laboratory. Fourteen additional participants were excluded because they failed an attention check item (i.e., “please select strongly disagree”).

### Procedure and materials

#### Iowa Netherlands Comparison Orientation Measure (INCOM)

We employed a Korean-translated version of the INCOM [20] validated in previous research [21]. The INCOM measures individual differences in people’s tendencies to engage in social comparisons of abilities (6-items; e.g., “I often compare myself with others with respect to what I have accomplished in life”) and opinions (5-items; e.g., “If I want to learn more about something, I try to find out what others think about it”). Participants rated the items using a 5-point scale (1 = *disagree strongly* to 5 = *agree strongly*). Individuals scoring higher on the INCOM tend to spend more time engaging in social comparisons [22]. Higher scores indicate greater tendencies to engage in social comparisons.

#### Personal Relative Deprivation

To measure PRD, we translated Callan et al.’s [3] 5-item PRDS-Revised into Korean. Korean translated versions of measures used in Studies 1 and 2 can be found in supporting information. Two translators both fluent in Korean and English took part in back-translation [23]. Participants indicated how strongly they agreed with each item given a 6-point scale (1 = *strongly disagree* to 6 = *strongly agree*). Higher scores indicate higher PRD.

#### Personality Traits

We also administered the Korean version of the Ten-Item Personality Inventory, which has been previously used and validated (TIPI; [24]; [25]). Participants indicated the extent to which they agreed with each statement given a 7-point scale (1 = *disagree strongly* to 7 = *agree strongly*).

#### Global physical health

Participants reported their physical health using a back-translated version of a single-item measure (“In general, would you say your physical health is”; [9]) given a 7-point scale (1 = *excellent* to 7 = very *poor*). This item was rescaled so that higher values indicate better global physical health.

#### Materialism

To measure materialism, the 9-item Material Values Scale (MVS; [26]) was back-translated by the authors. Previously, the back-translated Korean version of MVS has been shown to be cross-culturally applicable (Wong, Rindfleisch, & Rurroughs, 2003). The MVS assesses how much participants endorse materialistic values (e.g., “I admire people who own expensive homes, cars, and clothes”). Participants rated each item using a 7-point scale (1 = *strongly disagree* to 7 = *strongly agree*). Higher scores indicate higher materialism.

#### Income and education

Participants reported their annual household income before taxes given 11 categories (1,000,000 Korean won to 10,000,000 Korean won). Income responses were converted into estimates of absolute income using Parker and Fenwick’s [27] median-based estimator. Lastly, participants indicated their highest level of educational attainment given four categories (1 = *did not finish high school* to 4 = *postgraduate degree*). Korean-translated versions of all of the measures we employed can be found in the supporting information.

## Results

### Internal reliability and Exploratory Factor Analysis of the Korean PRDS

The PRDS showed low internal consistency (α = .38). In contrast to the single factor solution observed in previous research [3], an Exploratory Factor Analysis (EFA) of the Korean-translated PRDS (using principal axis factoring extraction with direct oblimin rotation) revealed two factors with eigenvalues greater than 1 (confirmed by visual inspection of the inflexion point of the scree plot), with the reversed scored items (2 and 4) loading onto a separate factor (eigenvalue = 1.26, 25.2% variance explained) than the remaining items (eigenvalue = 2.17, 43.38% variance explained; Table 1). The two factors were negatively correlated, *r* = -.21. Following Wong, Rindfleisch, and Burroughs’ [28] recommendations, Items 2 and 4 were therefore dropped from the Korean PRDS for subsequent analyses. The resulting 3-item PRDS had acceptable internal consistency (α = .75) and a simple factor structure from a follow-up EFA (eigenvalue = 2.00, 66.61% variance explained; no other eigenvalues > 1; Table 2).

**Table 1:** Summary of Exploratory Factor Analyses for the original Five-Item Korean-translated Personal Relative Deprivation Scale.

*Scale Items*	Study 1 (*N* = 224)			Study 2 (*N* = 186)			Study 1 and 2 Combined (*N* = 410)		
	Communalities and Rotated Factor Loadings								
	Com	Factor 1	Factor 2	Com	Factor 1	Factor 2	Com	Factor 1	Factor 2
1. I feel deprived when I think about what I have compared to what other people like me have.	.50	.69	-.07	.58	.76	-.01	.52	.70	-.07
2. I feel privileged compared to other people like me.	.35	-.19	.52	.52	-.56	.38	.42	-.34	.50
3. I feel resentful when I see how prosperous other people like me seem to be.	.53	.64	-.25	.53	.72	-.05	.53	.69	-.13
4. When I compare what I have with what others like me have, I realize that I am quite well off.	.36	.10	.61	.38	.03	.62	.35	.09	.60
5. I feel dissatisfied with what I have compared to what other people like me have.	.62	.80	.21	.73	.85	.27	.71	.85	.24
Eigenvalues		2.17	1.26		2.56	1.14		2.32	1.22
% of variance		43.48	25.20		51.12	22.81		46.43	24.34

^*a*^Items 2 and 4 were reverse-coded.

^b^Com = Extracted communality.

**Table 2:** Summary of Exploratory Factor Analyses for the revised Korean-translated Personal Relative Deprivation Scale (3-item).

*Scale Items*	Study 1 (*N* = 224)		Study 2 (*N* = 186)		Study 1 and 2 Combined (*N* = 410)	
	Communalities and Factor Loadings					
	Com	Loading	Com	Loading	Com	Loading
1. I feel deprived when I think about what I have compared to what other people like me have.	.52	.72	.55	.74	.50	.71
2. I feel resentful when I see how prosperous other people like me seem to be.	.46	.68	.55	.75	.53	.73
3. I feel dissatisfied with what I have compared to what other people like me have.	.51	.72	.65	.80	.60	.77
Eigenvalues		2.00		2.16		2.08
% of variance		66.61		72.12		69.42

^a^Com = Extracted communality

### Correlations and multiple regression analyses

Descriptive statistics, alpha reliabilities and inter-correlations among the focal measures are shown in Table 3.

**Table 3:** Descriptive statistics and inter-correlations for focal measures used in Studies 1 and 2.

Measures	Mean (*SD*)	1.	2.	3.	4.	5.	6.
**Study 1**							
1. INCOM-ability	3.31 (0.65)	(.80)					
2. INCOM-opinion	3.77 (0.58)	.53**	(.67)				
3. PRDS-3	2.70 (0.98)	.48**	.19**	(.75)			
4. Physical Health	4.63 (1.37)	-.10	.02	-.22**	--		
5. MVS-9	3.83 (1.05)	.39**	.10	.42**	-.06	(.83)	
**Study 2**							
1. INCOM-ability	2.74 (0.69)	(.59)					
2. INCOM-opinion	3.03 (0.72)	.44**	(.69)				
3. PRDS-3	3.03 (1.15)	.41**	.29**	(.81)			
4. Self-Esteem	3.10 (0.93)	-.12	-.05	-.21**	--		
5. Perceived Stress	3.22 (1.12)	.05	.07	.28**	-.12	--	
6. MVS-3	4.46 (1.33)	.22**	.20**	.33**	-.15*	.20**	(.80)

^*a*^INCOM = Iowa-Netherlands Comparison Orientation Measure; PRDS = Personal Relative Deprivation; MVS = Material Values Scale (3 or 9 items).

^b^Where applicable, alpha reliabilities are presented in parentheses along the diagonal.

^c^*p < .05. **p < .01.

The 3-item Korean PRDS correlated significantly positively with participants’ tendencies to engage in social comparisons of abilities and opinions. However, consistent with previous research with Western samples (e.g., [6]), a multiple regression analysis showed that social comparisons of abilities (*b* = .81, β = .53, se = .11), *t*(221) = 7.71, *p* < .001, but not opinions (*b* = -.16, β = -.10, se = .12), *t*(221) -1.38, *p* = .17, uniquely predicted PRD.

The 3-item Korean PRDS correlated significantly with materialism and self-rated health, such that participants higher in PRDS reported higher materialism and worse health. These associations were largely unchanged when controlling for age, gender, income, and education (PRDS with materialism: β = .42, *p* < .001; PRDS with health: β = -.18, *p* = .006).

Shown in Table 4, the three-item Korean PRDS scale correlated significantly with the Extraversion, Conscientiousness, Emotional stability, and Openness components of the TIPI. This pattern is largely consistent with the results of previous studies that measured PRDS and the TIPI among Western samples (see [3] and [29]). Specifically, the PRDS typically correlates negatively with Openness, Emotional Stability, and Conscientious, with more mixed findings across previous studies for Extraversion and Agreeableness. A full table of the correlations among all of the measures we employed in Study 1 can be found in the supporting information.

**Table 4:** Correlations between the Korean Personal Relative Deprivation Scale and components of the Ten-Item Personality Inventory.

	Personality Trait				
	*Extraversion*	*Agreeableness*	*Conscientiousness*	*Emotional Stability*	*Openness*
Korean 3-item PRDS	-0.25*	0.02	-.19*	-.31*	-.30*

^*a*^PRDS = Personal Relative Deprivation Scale.

^b^*p < .01.

### Mediation analyses

**Fig 1:**
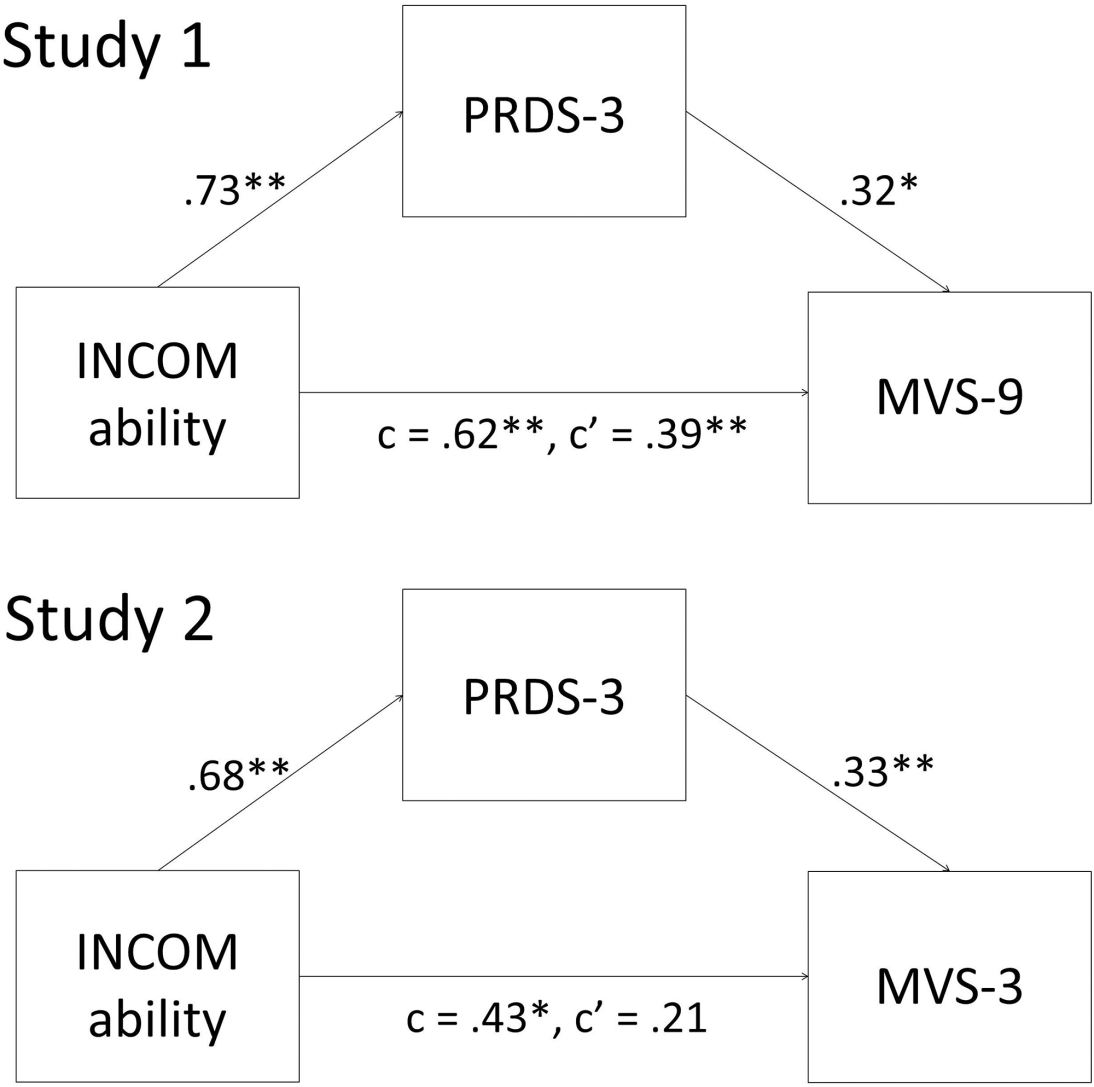
Mediation models for Studies 1 and 2. INCOM = Iowa-Netherlands Comparison Orientation Measure; PRDS = Personal Relative Deprivation Scale; MVS = Material Values Scale. Values depict unstandardized regression coefficients. ** *p* < .001, * *p* < .01.

Consistent with Kim et al. [6], bootstrapped mediation analyses ([30]; 10,000 resamples) revealed that the Korean PRDS mediated the relation between social comparison of ability and materialism (indirect effect = .232, 95% bias-corrected and accelerated confidence interval [BCa CI] of .119 and .372; Fig 1), suggesting that one of the reasons why tendencies to make social comparisons of abilities is associated with increased materialism is through personal relative deprivation.

## Study 2

The participants we recruited in Study 1 were mostly university students. In Study 2, we aimed to confirm and extend our Study 1 findings using a more representative Korean sample. Specifically, participants completed the 5-item Korean-translated PRDS (Table 1) along with measures of social comparison tendencies, self-esteem, perceived stress, and materialism. Based on previous findings using Western samples [3, 9], we expected that self-esteem would correlate negatively with PRD, and perceived stress would correlate positively with PRD. We also expected to replicate the Study 1 results that the tendency to make social comparisons of abilities but not opinions would predict PRD, and the positive relationship between social comparisons of abilities and materialism would be mediated by PRD.

## Method

### Participants

Participants (*N* = 186; 49% male; *M*_*age*_ = 34.36, *SD*_*age*_ = 10.41) were recruited by Nownsurvey, a widely used recruitment platform in South Korea (http://www.nownsurvey.com), to complete an online survey using a mobile device. Fourteen additional participants were removed for failing an attention check item.

### Procedure and measures

Participants completed a 6-item version of the INCOM [31], the PRDS as in Study 1, and a 3-item version of the MVS [26]. Self-esteem was assessed with a single item that we back-translated: “I have high self-esteem” [32]. Participants responded to this item using a 5-point scale (1 = *not very true of me*, 5 = *very true of me*). We used a back-translated single-item to assess perceived stress (“How much stress [e.g., because of hassles, demands] were you under recently?”; [33]), which participants responded to using a 5-point scale (1 = *felt very slightly or not at all*, 5 = *felt very much*). These single item scales were employed in previous research using Western samples and produced similar results to full item scales [9]. Participants also reported their household income as in Study 1.

## Results

### Internal reliability and Exploratory Factor Analysis of the Korean PRDS

The internal consistency of the 5-item PRDS was low (α = .31). An EFA as per Study 1 revealed two factors, with the two reverse-scored items (2 and 4) again loading onto a separate factor (eigenvalue = 1.14, 22.81% variance explained) from the remaining 3 items (eigenvalue = 2.56, 51.52% variance explained; Table 1). The two factors were negatively correlated, *r* = -.13. As in Study 1, we dropped these items from the PRDS. The resulting 3-item PRDS had acceptable internal consistency (α = .81) and a simple factor structure from a follow-up EFA (eigenvalue = 2.16, 72.12% variance explained; no other eigenvalues > 1; Table 2).

An EFA of the Study 1 and 2 data combined (Table 1) showed the same basic pattern as the individual studies, with items 2 and 4 loading onto a separate factor (eigenvalue = 1.22, 24.34% variance explained) than the remaining 3 items (eigenvalue = 2.32, 46.43% variance explained). The two factors were negatively correlated with the collated data, *r* = -.18. The resulting 3-item PRDS had acceptable internal consistency (α = .78) and a simple factor structure from a follow-up EFA (eigenvalue = 2.08, 69.42% variance explained; no other eigenvalues > 1; Table 2).

### Correlation and multiple regression analyses

Confirming the Study 1 results, a multiple regression analysis showed that social comparisons of abilities (*b* = .58, β = .35, se = .13), *t*(183) = 4.64, *p* < .001, but not opinions (*b* = .22, β = .14, se = .12), *t*(183) 1.83, *p* = .07, uniquely predicted PRD.

Consistent with previous research, the PRDS correlated negatively with self-esteem, and positively with materialism and perceived stress (Table 2). Multiple regression analyses showed that the associations between PRDS and stress and PRDS and materialism were unchanged when controlling for age, gender, income, and self-esteem (βs = .28 and .29, respectively, *ps* < .001) (cf. [6]; [9])

### Mediation analyses

Consistent with Study 1, bootstrapped mediation analyses revealed that the Korean PRDS mediated the relation between social comparison of ability and materialism (10,000 samples; indirect effect = .223, 95% BCa CI of .091 and .409; Fig 1).

## General discussion

Our goal was to develop and validate a Korean-translated version of Callan et al.’s [3] PRDS. The results of two studies showed that the 5-item Korean PRDS did not have a single-factor structure like the original scale [3]. In both studies, the two reverse-worded items loaded onto a separate factor than the remaining items. This pattern is perhaps not surprising, as reverse-worded items can weaken internal validity when translated into a different language, and the commonly accepted solution is to remove items that do not load on the primary factor [28]. Another reason for this discrepant pattern of loadings might have been difficulties with translation: our Korean participants might have interpreted the words “quite well off” (translated to “financially successful”) and “privileged” (translated to “sense of entitlement”) differently than their intended meanings in English.

Despite the full, 5-item Korean PRDS not showing a simple factor structure, the reduced 3-item version demonstrated good psychometric properties and convergent validity. Consistent with previous research using Western samples, a tendency to make social comparisons of abilities (but not opinions) uniquely predicted scores on the 3-item Korean PRDS, and higher PRD was associated with lower self-esteem, increased materialism, increased stress, and worse physical health (cf. [6]; [9]). Thus, our results suggest that at least some of the known antecedents and consequences of PRD previously observed among Western samples replicate in an Eastern cultural context. Given our findings, we recommend that researchers interested in investigating cross-cultural differences in PRD use the 3-item version across contexts. It is worth noting even in Western samples, the 3-item version of the PRDS we recommend here often performs as well as the full, 5-item version. For example, through a re-analysis of Callan et al.’s (2015; *N* = 397) Study 2 data, PRDS scores computed from the 3-items (Items 1, 3, and 5 from Table 1 above) and all 5-items (αs = .83 and .83, respectively) correlated with perceived stress to a similar extent (*r*s = .57 and .54, respectively). Similarly, re-analysis of Kim et al.’s (2017) Study 1 data showed that the 3-item and the 5-item PRDS (αs = .88 and .87, respectively) correlated with the MVS to a similar degree (*r*s = .53 and .49, respectively).

Our results support that, in East Asian samples, PRD might play a crucial role in explaining the relation between social comparison tendency and a variety of beliefs and behavior that are relevant to health and materialistic values. For instance, people accustomed to Eastern culture tend to show overly relation-oriented self-representation and this has a negative effect on subjective well-being [34]. Nevertheless, whether and how a greater tendency to make social comparisons relates to lower levels of subjective well-being in collectivistic culture is unclear. Given that East Asians also think of others’ financial success as an index of quality of life more so than Westerners do [35], PRD might be an important psychological construct in explaining this relationship. Future research should further clarify whether PRD mediates the effect of social comparison tendency, highlighted in Eastern culture, on subjective well-being and life satisfaction.

## Supporting information

S1 Table Inter-correlations between measures used in Study 1. (PDF)

S2 Table Inter-correlations between measures used in Study 2. (PDF)

S1 Appendix Korean versions of measures used in Studies 1 and 2. (PDF)
