# Supplementary material for: Development and preliminary validation of a Korean version of the Personal Relative Deprivation Scale
Source: PLoS One. 2018 May 10;13(5):e0197279. doi: 10.1371/journal.pone.0197279 (PMC5945005; doi:10.1371/journal.pone.0197279)
Supplement: S1 Table — (PDF) [file pone.0197279.s001.pdf]

S1 Table. Inter-correlations between Measures in Study 1.

Measures	*M* (*SD*)	1.	2.	3.	4.	5.	6.	7.	8.	9.	10.	11.	12.
1. PRDS-3	2.70 (.98)	(.75)											
2. INCOM- ability	3.31 (.65)	.48**	(.80)										
3. INCOM- opinion	3.77 (.58)	.19**	.53**	(.67)									
4. MVS	3.83 (1.05)	.42**	.39**	.10	(.83)								
5. Health	3.37(1.37)	-.22**	-.10	.02	-.06	--							
6. Income	6,548.61k (₩) (4,374.54.k)	.003	.07	.01	.10	-.006	--						
7. Education	2.25 (.53)	-.04	-.17*	-.21**	-.19**	-.08	-.04	--					
8. Extraversion	4.57 (1.40)	-.25**	-.13	.09	.08	.21**	.12	-.05	--				
9. Agreeableness	4.46 (1.07)	.02	.09	.03	.11	.08	.08	-.04	-.11	--			
10. Conscientiousness	4.11 (1.26)	-.19**	-.12	-.04	-.10	.26**	-.03	.07	-.04	.16*	--		
11. Emotional Stability	3.84 (1.39)	-.31**	-.32**	-.19**	-.13	.36**	.03	.13	-.06	.18**	.33**	--	
12. Openness	4.59 (1.29)	-.30**	-.22**	-.12	-.14*	.13*	.03	.03	.36**	-.28**	-.006	-.06	--

[a]PRDS-3 = Items 1, 3, and 5 from the Personal Relative Deprivation Scale; INCOM = Iowa Netherlands Comparison Orientation Measure; MVS = Material Values Scale; Health = Self-rated Global Physical Health.
[b]When applicable, alpha reliabilities are presented in parentheses along the diagonal.
[c]** $p < .01$, * $p < .05$.
