# Supplementary material for: Development and preliminary validation of a Korean version of the Personal Relative Deprivation Scale
Source: PLoS One. 2018 May 10;13(5):e0197279. doi: 10.1371/journal.pone.0197279 (PMC5945005; doi:10.1371/journal.pone.0197279)
Supplement: S2 Table — (PDF) [file pone.0197279.s002.pdf]

S2 Table. Inter-correlations between Measures used in Study 2.

Measures	*M* (*SD*)	1.	2.	3.	4.	5.	6.
1. PRDS-3	3.03 (1.15)	(.81)					
2. INCOM-ability-3	2.74 (.69)	.41**	(.59)				
3. INCOM-opinion-3	3.03 (.72)	.29**	.44**	(.69)			
3. MVS-3	4.46 (1.33)	.33**	.22**	.20**	(.80)		
4. Stress	3.22 (1.12)	.28**	.05	.07	.20**	--	
5. Self-Esteem	3.10 (.93)	-.21**	-.12	-.05	-.15*	-.12	--
6. Income	3,395.09k (₩) (2,424.27k)	.02	.06	.06	.14	-.11	.17*

[a]PRDS = Personal Relative Deprivation Scale; INCOM = Iowa Netherlands Comparison Orientation Measure (Schneider & Schupp, 2014); MVS = Material Values Scale.

[b]When applicable, alpha reliabilities are presented in parentheses along the diagonal.

[c]** $p < .01$, * $p < .05$.
