# Supplementary material for: Development and preliminary validation of a Korean version of the Personal Relative Deprivation Scale
Source: PLoS One. 2018 May 10;13(5):e0197279. doi: 10.1371/journal.pone.0197279 (PMC5945005; doi:10.1371/journal.pone.0197279)
Supplement: S1 Appendix — (PDF) [file pone.0197279.s003.pdf]

S1 Appendix. Korean Version of Measures used in Studies 1 and 2

S1-1 Appendix. Korean Version of Personal Relative Deprivation Scale.

Items

1. 나와 비슷한 사람들이 가진 것들을 내가 가진것과 비교해 볼 때 박탈감을 느낀다.
   [I feel deprived when I think about what I have compared to what other people like me have]

2. 나와 비슷한 사람들과 나를 비교할 때 특권의식을 느낀다.
   [I feel privileged compared to other people like me]

3. 나와 비슷한 사람들이 금전적으로 성공하는 것을 볼 때 원망스럽다.
   [I feel resentful what I see how prosperous other people like me seem to be]

4. 내가 가진 것들을 나와 비슷한 사람들이 가진 것들과 비교해 보면 사실 꽤 금전적으로 성공했다고 느껴진다.
   [When I compare what I have with what others like me have, I realize that I am quite well off]

5. 내가 가진 것들을 나와 비슷한 사람들이 가진 것들과 비교해 볼 때 불만족스럽다.
   [I feel dissatisfied with what I have compared to what other people like me have]

[a]Items 2 and 4 were reverse-coded.
[b]Participants were given a 6 point-scale (1= 전혀 동의하지 않는다 [strongly disagree], 2 = 동의하지 않는다 [disagree], 3 = 약간 동의하지 않는다 [somewhat disagree], 4 = 약간 동의한다 [somewhat agree], 5 = 동의한다 [agree], 6 = 매우 동의한다[strongly agree]).
[c]Original items are presented in brackets.

S1-2 Appendix. Korean Version of Measure for Self-Rated Global Physical Health.

일반적으로 생각해 볼 때, 본인의 신체적 건강이 어떤 상태라고 할 수 있을까요?
[In general, my health is……?]

1. 아주 훌륭하다 [Excellent]
2. 매우 좋다 [very good]
3. 좋다 [good]
4. 그럭저럭 괜찮다 [somewhat good]
5. 나쁘지 않은 편이다 [fair]
6. 좋지 않다 [poor]
7. 매우 좋지 않다 [very poor]

S1-3 Appendix. Korean Version of Material Values Scale.

Items

---

1. 현재 나는 비싼 집, 차, 옷을 소유하고 있는 사람들을 동경한다.
   [I admire people who own expensive homes, cars, and clothes]

2. 내가 지금 소유하고 있는 것들이 내가 인생을 얼마나 잘 살고 있는지 보여준다고 생각한다.
   [I feel like the things I own say a lot about how well I'm doing in life)

3. 현재 나는 사람들에게 깊은 인상을 심어줄 수 있을 소유물을 가졌으면 하고 바란다.
   [I like to own things that impress people]

4. 나는 소유물들을 간단하게 유지하는 인생을 살고 싶다.
   [I'd rather keep life simple, as far as possessions are concerned]

5. 최근 무언가를 사는 것은 나를 꽤 행복하게 만드는 것 같다.
   [I feel that buying things would give me a lot of pleasure]

6. 현재 나의 인생에 호화로운 것들이 많았으면 좋겠다.
   [I'd like a lot of luxury in my life]

7. 현재 내가 가지지 못한 것들을 가졌다면 내 인생이 더 나았을 거라는 생각이 든다.
   [I feel that my life would be better if I owned certain things I don't have]

8. 현재 내 형편에 살 수 없는 것들을 지금 살 수 있었다면 더 행복했을 것이다.
   [I'd be happier if I could afford to buy more things]

9. 현재 나는 내가 사고 싶은 것들을 살 형편이 못되어 상당히 괴롭다.
   [It bothers me quite a bit that I can't afford to buy all the things I'd like]

---

[a]Item 4 was reverse-coded.
[b]Participants were given a 7 point-scale (1= 전혀 동의하지 않는다 [strongly disagree], 2 = 동의하지 않는다 [disagree], 3 = 약간 동의하지 않는다 [somewhat disagree], 4 = 중립[neither agree nor disagree], 5 = 약간 동의한다 [somewhat agree], 6 = 동의한다 [agree], 7 = 매우 많이 동의한다 [strongly agree]).
[c]Original items are presented in brackets.

S1-4 Appendix. Korean Version of Measure for Income.

가정의 월 평균 소득 (세전)은 얼마입니까? 월평균 소득은 집에서 돈을 벌고 있는 모든 사람의 소득을 합하여 12 로 나눈 월평균 금액을 말합니다 (결혼 후 분가한 형제, 자매 제외). 다음 보기 중 하나를 선택해 주십시오.

[What is your monthly household income before tax? Monthly income includes all family members' (excluding those who have separate households) annual income divided by 12. Please select one of the following categories]

1. 월 100 만원 미만 [below 1 million won per month]
2. 월 100 만원 이상 – 200 만원 미만 [1 million won or over – 2 million won per month]
3. 월 200 만원 이상 – 300 만원 미만 [2 million won or over – 3 million won per month]
4. 월 300 만원 이상 – 400 만원 미만 [3 million won or over – 4 million won per month]
5. 월 400 만원 이상 – 500 만원 미만 [4 million won or over – 5 million won per month]
6. 월 500 만원 이상 – 600 만원 미만 [5 million won or over – 6 million won per month]
7. 월 600 만원 이상 – 700 만원 미만 [6 million won or over – 7 million won per month]
8. 월 700 만원 이상 – 800 만원 미만 [7 million won or over – 8 million won per month]
9. 월 800 만원 이상 – 900 만원 미만 [8 million won or over – 9 million won per month]
10. 월 900 만원 이상 – 1,000 만원 미만 [9 million won or over – 10 million won per month]
11. 월 1,000 만원 이상 [10 million won or over per month]

S1-5 Appendix. Korean Version of Measure for Education.

학력 [Education]

1. 고등학교를 마치지 않았다. [Did not finish high school]
2. 고등학교를 졸업했다. [High school graduation]
3. 대학교를 졸업했다. [College graduation]
4. 대학원을 졸업했다. [Postgraduate degree]

S1-6 Appendix. Korean Version of 1-item Self-Esteem Measure.

아래 문장을 읽고 해당되는 정도를 아래 숫자들로 표시해 주십시오.
"나는 높은 자존감을 가졌다." [I have high self-esteem]

1. 전혀 그렇지 않다 [not very true of me]
2.
3.
4.
5. 매우 그러하다 [very true of me]

S1-7 Appendix. Korean version of 1-item Stress Measure.

최근에 얼마나 스트레스를 받았나요? (예를 들어 번거로운 일들이나 혹은 부담 때문에 등등) [How much stress (e.g., because of hassles, demands) were you under recently?]

1 아주 조금 또는 전혀 받지 않았다 [felt very slightly or not at all]
2
3
4
5 매우 많이 받았다. [felt very much]
